# Distinct Allelic Patterns of Nanog Expression Impart Embryonic Stem Cell Population Heterogeneity

**DOI:** 10.1371/journal.pcbi.1003140

**Published:** 2013-07-11

**Authors:** Jincheng Wu, Emmanuel S. Tzanakakis

**Affiliations:** 1 Department of Chemical and Biological Engineering, State University of New York at Buffalo, Buffalo, New York, United States of America; 2 Department of Biomedical Engineering, State University of New York at Buffalo, Buffalo, New York, United States of America; 3 New York State Center of Excellence in Bioinformatics and Life Sciences, Buffalo, New York, United States of America; 4 Western New York Stem Cell Culture and Analysis Center, State University of New York at Buffalo, Buffalo, New York, United States of America; Northeastern University, United States of America

## Abstract

Nanog is a principal pluripotency regulator exhibiting a disperse distribution within stem cell populations in vivo and in vitro. Increasing evidence points to a functional role of Nanog heterogeneity on stem cell fate decisions. Allelic control of Nanog gene expression was reported recently in mouse embryonic stem cells. To better understand how this mode of regulation influences the observed heterogeneity of NANOG in stem cell populations, we assembled a multiscale stochastic population balance equation framework. In addition to allelic control, gene expression noise and random partitioning at cell division were considered. As a result of allelic Nanog expression, the distribution of Nanog exhibited three distinct states but when combined with transcriptional noise the profile became bimodal. Regardless of their allelic expression pattern, initially uniform populations of stem cells gave rise to the same Nanog heterogeneity within ten cell cycles. Depletion of NANOG content in cells switching off both gene alleles was slower than the accumulation of intracellular NANOG after cells turned on at least one of their Nanog gene copies pointing to Nanog state-dependent dynamics. Allelic transcription of Nanog also raises issues regarding the use of stem cell lines with reporter genes knocked in a single allelic locus. Indeed, significant divergence was observed in the reporter and native protein profiles depending on the difference in their half-lives and insertion of the reporter gene in one or both alleles. In stem cell populations with restricted Nanog expression, allelic regulation facilitates the maintenance of fractions of self-renewing cells with sufficient Nanog content to prevent aberrant loss of pluripotency. Our findings underline the role of allelic control of Nanog expression as a prime determinant of stem cell population heterogeneity and warrant further investigation in the contexts of stem cell specification and cell reprogramming.

## Author Summary

Nanog is a key factor influencing the decision of a stem cell to remain pluripotent or differentiate. Each embryonic stem cell (ESC) in a population exhibits fluctuating Nanog levels resulting in heterogeneity which affects cell fate specification. The allelic regulation of Nanog was demonstrated recently but its implications on population heterogeneity are unclear. We developed a multiscale population balance equation (PBE) model and compared our results with pertinent experimental studies. Under allelic control the profile of Nanog features three peaks or distinct states. Transcriptional noise causes the distribution to become bimodal as suggested previously. When stem cells carrying a reporter transgene in an allelically regulated locus were examined, we observed non-matching distributions of the endogenous and reporter proteins. This led us to investigate the performance of reporter systems depending on insertion of the transgene in one or both alleles and the protein degradation dynamics. Lastly, our model was employed to address how allelic regulation affects the maintenance of pluripotency in stem cells with a single Nanog allele deletion. A fraction of these cells remains pluripotent while deletion of a single allele does not simply reduce NANOG uniformly for all ESCs but modulates NANOG heterogeneity directly.

## Introduction

Nanog is a principal pluripotency regulator of embryonic stem cells (ESCs) in the early blastocyst. Mouse ESC (mESC) self-renewal is supported by Nanog in the absence of leukemia inhibitory factor (LIF) [1], [2] while Nanog knockdowns experience changes in global gene expression and loss of pluripotency [3]–[5]. In human ESCs (hESCs), Nanog reduction or overexpression leads to differentiation or inhibition of lineage commitment, respectively [6], [7]. Growth factors such as basic FGF and activin A known to maintain the pluripotency of hESCs target Nanog [8]–[10] further illustrating its prominent role in the decision of stem cells to self-renew or differentiate.

Single ESCs in vivo and in vitro exhibit fluctuating levels of several markers [11], [12] including Nanog [13], [14], which appears to regulate the heterogeneity of stem cell populations through feedback mechanisms with other transcription factors [15]. A bimodal distribution of Nanog has been reported in mESCs and hESCs carrying a reporter gene encoding the green fluorescence protein (GFP) in the Nanog gene locus [16], [17]. These observations have prompted the development of mathematical models to gain further insights into the mechanisms underlying Nanog heterogeneity. Nanog dynamics depicted in gene regulatory networks (GRNs) featuring feedback loops with transcriptional partners (mainly Oct4 and Sox2), are elicited via excitability [18] or oscillatory patterns [19]. According to these models, stem cells shuttle between a pluripotent Nanog^high^ state and a differentiation-permissive Nanog^low^ or Nanog^−^ state [14], [18]. Cells from the latter state reestablish the bimodal distribution under non-differentiating conditions pointing to the robustness of Nanog expression heterogeneity.

However, recent findings of allelic regulation of Nanog expression in mESCs shine new light on the observed heterogeneity of stem cell populations with respect to their Nanog profile. In an elegant study, Miyanari et al. [20] showed that cells in early pre-implantation mouse embryos express Nanog monoallelically but transition to a biallelic pattern in the late blastocyst. Similarly, mESCs cultured under typical maintenance conditions with LIF and serum express Nanog from a single allele whereas most mESCs treated with GSK3 and MAP inhibitors (2i condition [21]) activate both Nanog alleles. Accordingly, four distinct subpopulations of mESCs are observed depending on whether Nanog is expressed from each of the two alleles, both or none. This suggests that allelic regulation is a previously unaccounted source of stem cell population heterogeneity with the Nanog distribution comprising three cell groups (i.e. with monoallelic, biallelic and no expression of the gene).

This seemingly contradicts previous studies reporting only two groups (Nanog^low^ and Nanog^high^) of cells and calls for the development of a new framework incorporating the recent findings on the allelic control of Nanog expression. In fact, the two-state Nanog expression view of mESCs was recently reassessed and an intermediate state (middle Nanog) was added but without accounting explicitly for the allelic expression of the gene [22]. Moreover, previous models yielded fluctuations in Oct4 as coincident with those in Nanog. Yet, Miyanari et al. [20] noted that Oct4 does not experience the same allelic regulation with Nanog illustrating further the need for reexamining current rationales proposed for the variability of Nanog in stem cell ensembles. The allelic control of Nanog expression also necessitates the reinterpretation of work utilizing mESC and hESC lines carrying a reporter gene (typically that of the green fluorescent protein or *gfp*) knocked in one of the two Nanog alleles (e.g. [16], [18]).

We assembled a multiscale stochastic population balance equation (PBE) model to investigate how the recently discovered allelic control of Nanog expression affects ESC population heterogeneity. The distribution of pluripotency regulators in stem cell ensembles is determined by multiple processes transpiring at different physical and temporal scales. In addition to its allelic regulation, Nanog interacts with several other known and still unidentified factors and signals at the molecular level in a stochastic fashion. However, events at the cellular level (e.g. mitosis) also affect the content of Nanog and its partners in self-renewing stem cells [23]. Here allelic regulation was incorporated in our model to describe the distribution of Nanog in self-renewing mESCs [20]. Most notably, the newly observed regulatory mechanism is shown to be sufficient to give rise to a multimodal distribution of Nanog in a stem cell ensemble even in the absence of transcriptional noise. Pitfalls stemming from the use of Nanog reporter cell lines are demonstrated indicating the value of the PBE framework as a tool aiding in the (re)interpretation of the data from such experiments. Finally, quantitative analysis was performed of the Nanog signature of *Nanog* ^+/−^ mutant ESCs, the capacity of which to maintain their pluripotent state has been debatable. Our data explicates how these stem cells maintain a group of self-renewing cells through allelic control despite their higher propensity for differentiation and lower average content of NANOG protein.

## Results

### Model development

The model was developed in three stages. First, a linear system was constructed describing the temporal evolution of the mESC population achieving dynamic equilibrium. Cell proliferation kinetics and transition rates between different subpopulations of allelic Nanog expression were determined based on data from Miyanari and Torres-Padilla [20]. Then, a single-cell model was assembled for Nanog gene expression. Finally, a system of PBEs was casted linking the single-cell to the population dynamics.

### Proliferation and transition kinetics

**Figure 1:**
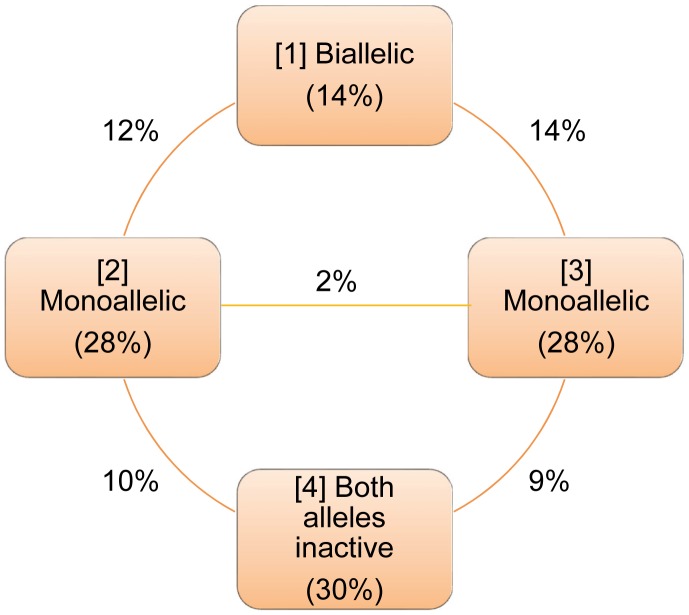
Dynamic equilibrium among groups of self-renewing mESCs exhibiting different patterns of allelic expression of *nanog*. The fraction of the mESC population residing in each group is noted in parentheses. Similarly, cells transitioning between two groups are represented by the number (percentage) shown between these groups. Cells switch between patterns of allelic Nanog expression in intervals equivalent to multiples of the cell cycle time.

Mouse ESC types were defined (Figure 1) depending on whether *nanog* is expressed from both alleles (type 1) simultaneously or from a single allele (types 2 and 3) while there are also cells with both alleles being inactive (type 4).

**Figure 2:**
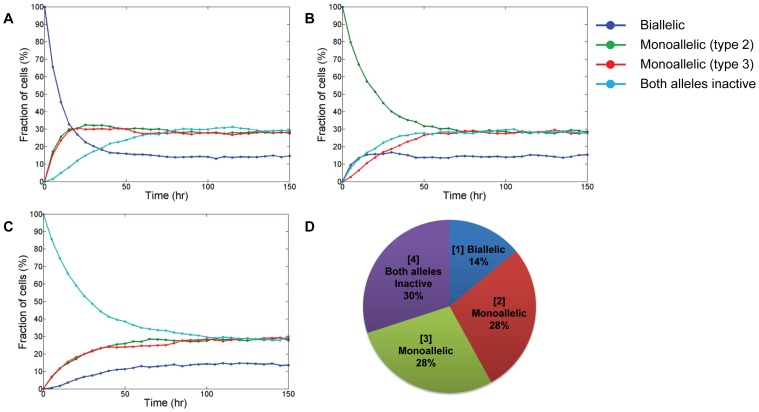
PBE model prediction of the time evolution of a mESC population starting with cells which exhibit a uniform pattern of allelic Nanog expression. Initially, all cells have: (A) Both alleles active (type 1), (B) both alleles inactive (type 4), or (C) only a single allele active (type 2 or type 3). (D) Composition at equilibrium of the mESC population based on their allelic expression of Nanog.

The percentage of each subpopulation was calculated based on data from mESCs cultured with leukemia inhibitory factor (LIF) [20]. Briefly, approximately 30% of the mESCs were NANOG^−^ (type 4) as determined from immunocytochemisty data for a total population of 135 cells (suppl. Figure 4b in reference [20]). Of the remaining 70% of (NANOG^+^) mESCs, roughly 80% expressed the gene monoallelically and 20% from both alleles (Figure 2e in reference [20]). Hence, 56% (types 2 and 3) and 14% (type 1) of the mESCs expressed *nanog* from a single and both alleles, respectively. Because no bias was reported for *nanog* expression from a specific allele, one can assume that each of types 2 and 3 comprises 28% of the total mESC population.

Single-cell allele-specific RT-PCR results were also provided in the same report (suppl. Figure 3b in Miyanari et al. [20]). Out of 19 mESCs examined, four cells were biallelic, ten cells were monoallelic and the remaining were classified as type 4 cells corresponding to the following fractions: 21.1% of type 1, 52.6% of types 2 and 3 and 26.3% of type 4. This population composition was close to that derived from the immunocytochemistry and RNA FISH data. However, the mESC fraction values calculated based on immunocytochemistry/RNA FISH were preferred due to the significantly larger sample size compared to that in the allele-specific RT-PCR experiment.

The stochastic switching of mESCs from one allelic pattern of *nanog* expression to another can be modeled as a time homogeneous Markov chain with four states (Figure 1). Cells switching satisfies the Markov property that the future state of each cell depends only on its current state. The fractions of cells per state at equilibrium are the elements of the limiting (equilibrium) distribution of the chain 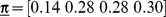.

The transition matrix 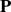 can be calculated from the percentages of the mESC population shuttling between states (see the **Materials and Methods** section):
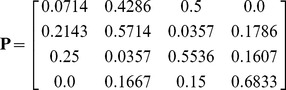
(1)satisfying the condition: 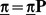. The transition rates 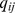 between states i and j provide information regarding the kinetics of the process and these can be calculated from the transition probabilities 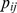 (see **Materials and Methods**) taking into account that the fractions of mESCs in each state and between states have been determined over a single cell cycle T_d_ or about 10 hours (suppl. Figure 6 in reference [20]). This yields the transition rate matrix:
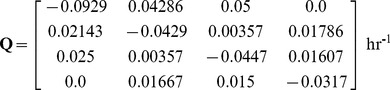
(2)with 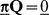.

In addition, the proliferation rate 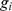 of cells in the i^th^ state can be calculated based on the doubling time T_d_ of the mESC population. All mESCs in the population have the same proliferation kinetics regardless of the allelic regulation of *Nanog* expression:
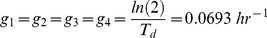
(3)


The mESC population can be described by a row vector 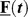 with four elements representing the number of mESCs of each type (i.e. F_1_(t), F_2_(t), F_3_(t), F_4_(t)). Taking an exponential growth for the mESC population, the vector 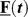 satisfies the equation
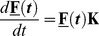
(4)


The matrix 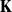 is the sum of the transition rate matrix 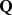 and a diagonal matrix with the growth rates 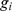 of mESCs belonging to the four types, i.e.
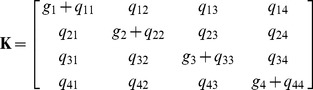
(5)


Each subpopulation can also be described by a percentage Z_i_(t) so that F_i_(t) = Z_i_(t)F_t_(t) (F_t_(t): total cell number). Then, Equation 4 can be re-casted (see **Materials and Methods**):
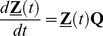
(6)with a stationary distribution 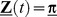 when 
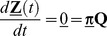
.

### Single-cell gene expression model

After calculating the proliferation rate and kinetics of transitioning between subgroups with different allelic expression of *nanog*, a model was constructed for single-cell level gene expression. A set of differential equations can be written encompassing the active (“On”) and inactive (“Off”) states for each allele (Table 1). The production and degradation dynamics of Nanog protein per allele are represented by zeroth and first order kinetics, respectively. The same kinetics were assumed for both Nanog alleles.

**Table 1:** Summary of mESC subtypes based on the state of each Nanog allele.

Cell type	NANOG allele 1	NANOG allele 2
**1**	On	On
**2**	On	Off
**3**	Off	On
**4**	Off	Off

Cell types 1–4 are shown in Figure 1.

For the j^th^ Nanog allele (j = 1, 2) we write:
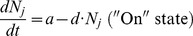
(7)

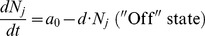
(8)


Noise is an integral part of the transcription of genes [24] and can be taken into account by representing gene expression dynamics with stochastic differential equations (SDEs). To that end, we also employed the SDEs below to describe Nanog expression:
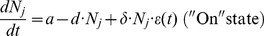
(9)

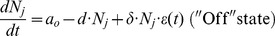
(10)


The noise terms 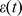 were taken as temporally uncorrelated, statistically independent unit Gaussian white noise. The term 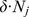 refers to the intensity of the noise linearly related to NANOG [19]. Solutions to the SDEs were obtained via the Euler-Maruyama method [25].

The half-life (t_1/2_) of the NANOG protein in mESCs was experimentally determined to be approximately 2 hours [26], [27], or
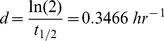
(11)


The production rate of NANOG protein per allele is estimated at 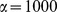 molecules/hr. The Nanog^high^ population was considered as comprising cells producing Nanog biallelically and monoallelically at a ratio of 1∶4 [20]. Then, this value of 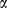 yields approximately 3,700 NANOG molecules per *nanog*-expressing mESC (Nanog^high^) on average at steady state [18]. Flow cytometry analysis of Nanog expression in mESCs reveals that the means of the Nanog^low^ and Nanog^high^ states are different by two orders of magnitude [16], [18]. Consequently, the rate of NANOG protein generation from the “off” state allele is set at 1% of the “on” rate i.e. 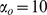 molecules/hr. Hence, even mESCs with both alleles at the ‘off’ state exhibit a baseline of NANOG expression. It should be noted however, that varying the values of 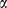 and 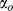 by 20% did not alter the modeling results qualitatively (Figure S1). For case studies involving Nanog reporter systems (e.g. with GFP expression), Equations 7 and 8 were utilized to describe the expression of the reporter protein with the same values for 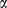 and 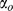 as above. The degradation rate 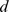 however, was calculated based on the t_1/2_ of the reporter protein. A t_1/2_ of 20 hours is reported for GFP [28].

### Multiscale cell PBE model

With the elements of 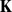 determined for mESC proliferation and transition between patterns of allelic Nanog expression and the single-cell gene expression model in place, we proceeded to construct a PBE-based system to describe, analyze and predict the effects of allelic regulation on the NANOG heterogeneity of stem cell populations. This framework takes into account processes such as gene expression and division occurring at the single-cell and population levels and spanning multiple time scales. The framework below comprises four PBEs, i.e. one PBE for each of the four distinct mESC groups based on the allelic expression of Nanog.
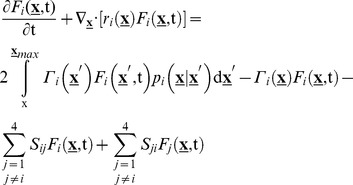
(12)


The integers i and j refer to the mESC type (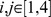) with i≠j and 
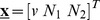
.

The state vector variables *N_1_* and *N_2_* correspond to NANOG levels originating from each one of the two alleles and *v* represents the cell size (volume) indicative of the cell's division potential [29]. The growth rate of cell size is proportional to cell size as detailed previously [23] (see also **Materials and Methods**). The rates for Nanog expression (i.e. 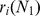 and 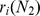) have been derived above (Equations 7–10). The dividing rate 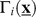 and partitioning function 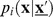 have been reported previously for stem cells [23] and details are provided in the **Materials and Methods** section. In addition, the allelic switching rates 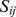 correspond to the transition rates 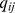 (Equation 2), i.e.:
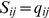
(13)


Numerical solutions of the PBE system were obtained via a stochastic kinetic Monte Carlo algorithm [23], [30] as described in **Materials and Methods**. This entails the calculation of the time between successive cell divisions and allelic switching (interval of quiescence) which is considered a Markov process.

### Allelic regulation contributes to a multimodal nanog profile in stem cell populations

According to the findings of Miyanari et al. [20], mESCs achieve an equilibrium state as a composite of four subpopulation. This is reflected in the non-trivial solution 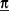 of Equation 6. Thus, we first examine if stem cells from each subpopulation can reconstitute the blended population at equilibrium in PBE model simulations. For this purpose, the starting population was set to 100% of single type mESCs and its temporal trajectory was tracked (Figure 2). Indeed, mESCs attained the same equilibrium state 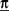 within 50–100 hr from initial ensembles of cells with a uniform *nanog* expression pattern. Thus, the allelic control of *nanog* allows mESCs to restore the population with constant fractions of different types at equilibrium.

**Figure 3:**
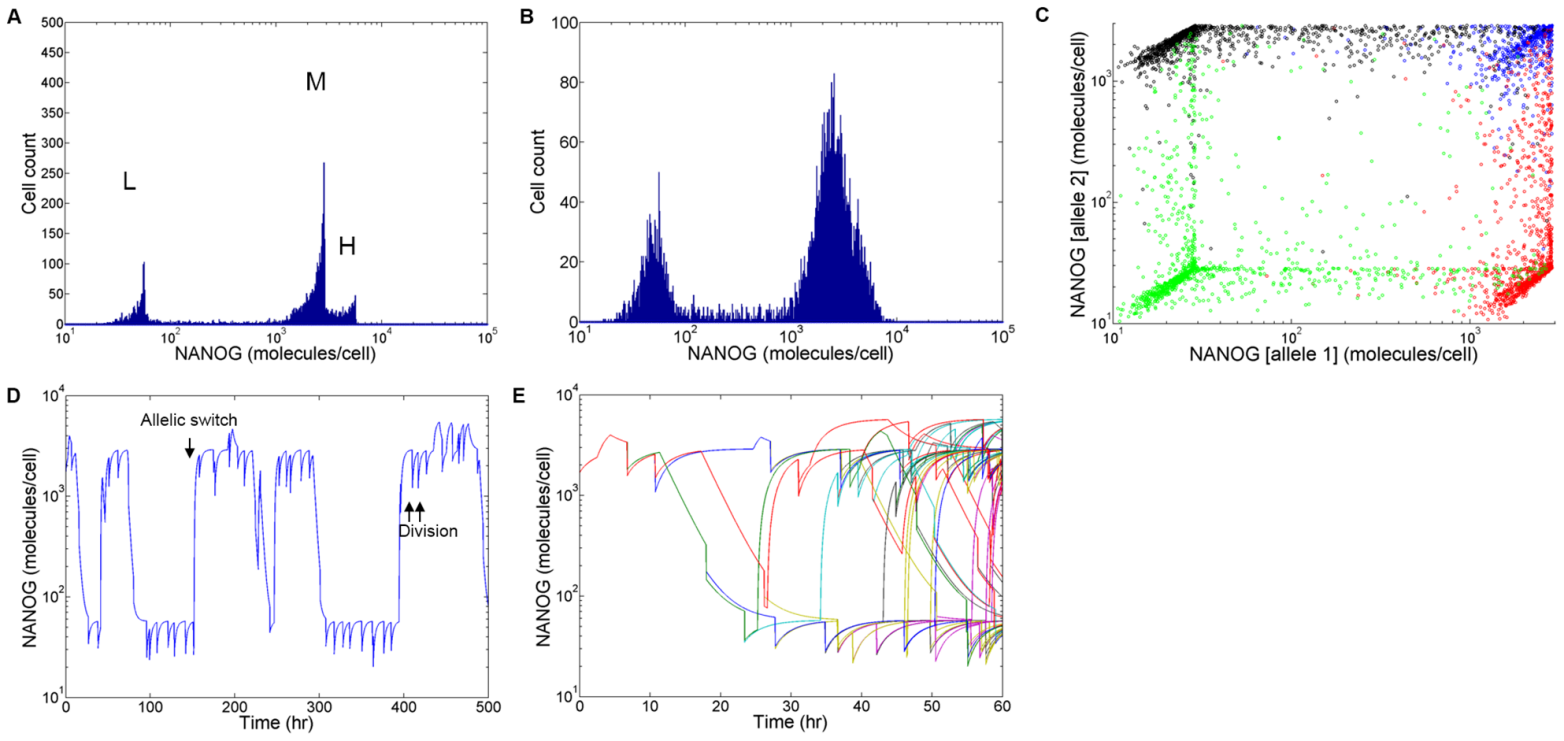
NANOG dynamics for mESC populations and single cells. (A) Overall NANOG expression distribution in equilibrium. Three distinct peaks of low (‘L’), middle (‘M’) and high (‘H’) NANOG content are observed. (B) The distribution of NANOG at equilibrium taking into account transcriptional noise. (C) A map of the NANOG content of each cell in the population. Different colors represent the four patterns of allelic Nanog expression. (D) Fluctuations in NANOG by a randomly selected single mESC. After each division only one daughter cell is shown. Arrows mark divisions and allelic switch events. (E) Starting with a single mESC expressing Nanog monoallelically, the resulting population after 60 hours contains cells of all four types. Both daughter cells were shown after each cell division. The trajectory of one daughter cell is denoted with the same color as the mother cell.

We then set out to investigate the extent to which allelic gene regulation contributes to NANOG expression macro-heterogeneity utilizing the PBE representation of the mESC population. Simulation results yielded three distinct peaks in the distribution of NANOG (Figure 3A). The NANOG^high^ region comprised mESCs with biallelic Nanog expression (peak ‘H’ in Figure 3A) and the NANOG^low^ area (peak ‘L’) contained cells with both alleles being inactive. These type ‘4’ cells may exhibit low levels of NANOG from leaky expression and from protein produced before entering this state (see below). In a flow cytometry assay, these cells may fall within the region of autofluorescence [18] or isotype control [23].

Notably, there is also a prominent third peak (‘M’) corresponding to mESCs with monoallelic *nanog* expression. The results seem to contrast the bimodal distributions of NANOG which have been reported for mESCs and hESCs carrying the *gfp* gene in their Nanog gene locus [16], [17]. We considered that noise associated with gene transcription and translation [31]–[33] may have an additional impact on NANOG variability. To test this, we employed SDEs (Equations 9–10) instead of deterministic ODEs (Equations 7–8) to depict the dynamics of Nanog. As shown in Figure 3B, a 20% noise relative to the NANOG protein level was sufficient for peaks ‘M’ and ‘H’ to merge yielding a bimodal profile. Potentially, other sources of noise during measurement (e.g. instrument noise) may also contribute to the dispersion in NANOG distribution effectively reducing the threshold of intrinsic noise in gene expression leading to the same two- (instead of three-) peak profile. In addition to stem cell lines expressing a reporter gene from the Nanog locus, stem cells stained with appropriate Nanog antibodies exhibit the same two-peak NANOG profile shown in Figure 3B after flow cytometry. Since our focus in this study was on how allelic regulation affects Nanog presentation in stem cell populations and fundamental experiments for quantification of intensity from other noise sources are still lacking, we considered the dynamics of Nanog as deterministic (Equations 7–8) in subsequent simulations.

The NANOG content of individual cells displaying different modes of allelic control was examined. For any snapshot of the mESC ensemble, four distinct subpopulations could be identified clustered approximately on the vertices of a square. Each vertex corresponded to mESCs with a specific pattern of allelic Nanog expression (Figure 3C). There were also cells transitioning between subgroups as indicated by their presence on the edges and inside the square area. Upon closer observation, a number of cells classified as type ‘4’ (green dots) were close to the vertex of cells expressing Nanog biallelically. Direct transition between types ‘1’ and ‘4’ was ruled out as it was not observed in experiments [20].

Another possible explanation is that cells having both of their Nanog alleles inactive which mapped close to the subgroup ‘1’ region most likely entered state ‘4’ only recently. Hence, their high Nanog content is mainly due to past elevated expression of *nanog*. This prompted us to investigate NANOG fluctuations in randomly selected single mESCs (Figure 3D) and the time required to build or deplete NANOG after allelic pattern switching. Division and allelic switching events transpired stochastically at timescales longer than the periods for production and degradation of the protein. When the NANOG levels of all daughter cells derived from a single mESC are plotted over 60 hours (Figure 3E), a state for the population can be clearly seen as in Figure 3A. A cell with high Nanog content reverts to a state of minimal intracellular Nanog 15–20 hours after turning off expression from both alleles. This explains the presence of type ‘4’ cells in the vicinity of the type ‘1’ cell vertex and along the ‘1’–‘2’ and ‘1’–‘3’ type edges (Figure 3B). In contrast, type ‘4’ cells switching to monoallelic Nanog expression build their Nanog content reaching higher levels faster (less than 5 hours). This explains the presence of only few type ‘2’ and ‘3’ cells close to the type ‘1’ vertex.

Altogether, the allelic regulation of Nanog expression leads to macro-heterogeneity of the population with the assortment of cells into three groups which become less distinct under the influence of noise. The population and its Nanog profile can be reconstituted from individual self-renewing mESCs despite differences in their Nanog expression patterns. Interestingly enough, the time for depletion of intracellular NANOG reserves by stem cells switching to the state of ‘no-active allele’ expression is significantly longer than for reaching a high NANOG content after cells enter states of monoallelic gene expression from state ‘4’.

### Monitoring target protein expression in reporter stem cell lines: Effects of allelic regulation and target/reporter protein half-lives

The difference in the lag for adjustment of NANOG content after switching to a particular state of allelic Nanog expression is a function of parameters such as the cell T_d_, the average frequency of switching between patterns and the t_1/2_ of the protein. Whereas the first two parameters depend on the cell type, the latter is also largely specific to the protein of interest. This consideration is pertinent to mESC and hESC reporter lines, which are increasingly utilized in stem cell research. In these cells, a reporter gene such as *gfp* is knocked in one of the Nanog allelic loci [16], [17]. With this design, GFP and NANOG are assumed to have the same production rate [34] lending credence to the notion that GFP should track NANOG closely.

Nevertheless, two potential issues arise with such design. First, differences in the reporter and endogenous protein degradation kinetics may also effect divergence in the profiles of the two gene products. The GFP has a longer t_1/2_ (∼20 hours [28]) than that of NANOG (∼2 hours). Second, the existence of allelic regulation suggests that a reporter gene expressed from one allele via the promoter of a native gene may not be representative of the overall level of the target gene. These concerns prompted a more detailed investigation of the potential disparity in the expression of the reporter gene and endogenous NANOG subjected to allelic control.

**Figure 4:**
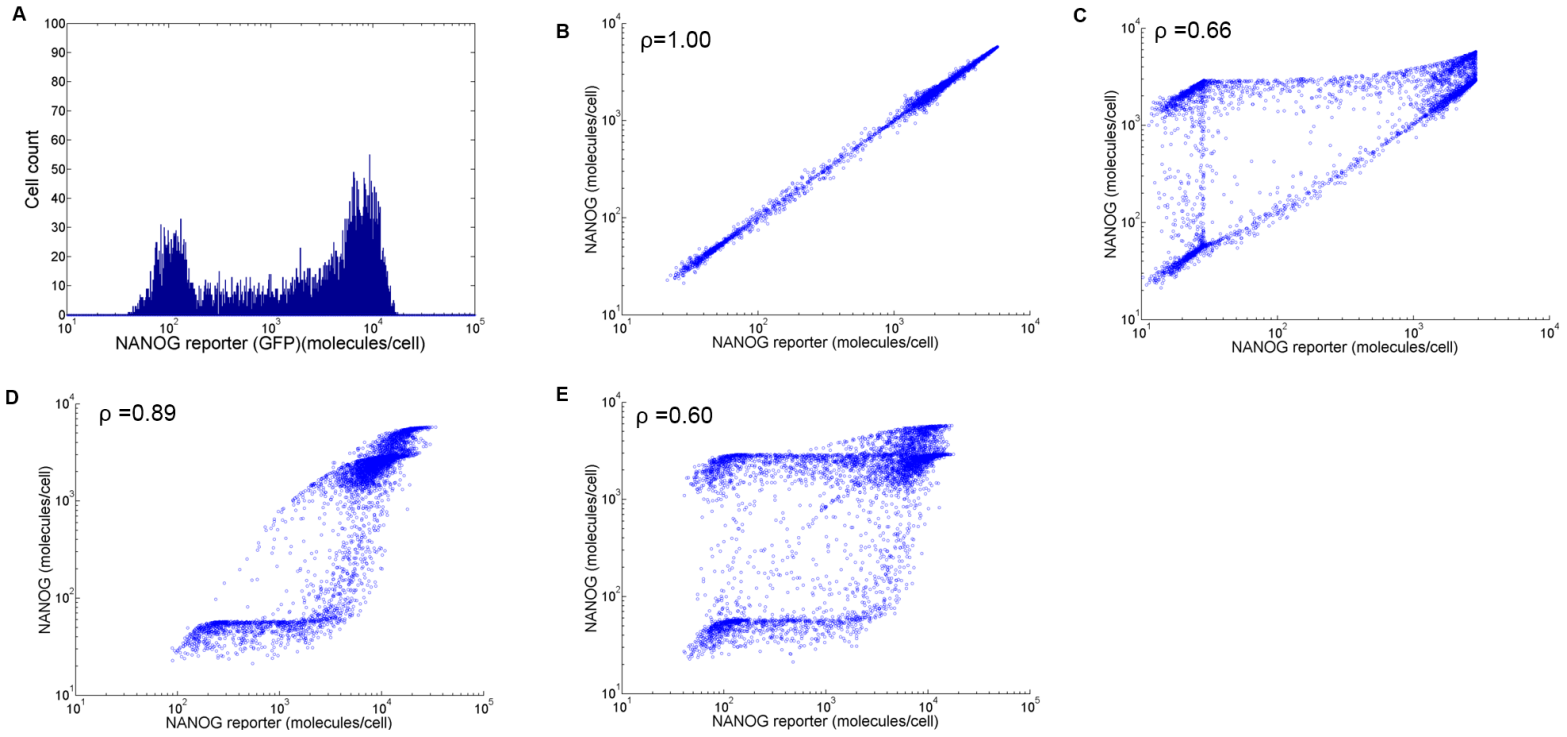
Reporter and endogenous NANOG protein levels under different conditions. (A) Observed NANOG reporter (GFP) expression level in stem cells with a single-allele insertion of the *gfp*. The expression levels of NANOG and the reporter are shown assuming the same t_1/2_ (2 hours) for the reporter and NANOG and the reporter gene inserted in (B) both alleles, or (C) one allele. NANOG and reporter levels are also shown for t_1/2(NANOG)_ = 2 and t_1/2(GFP)_ = 20 hours with the reporter gene inserted in (D) both, or (E) a single allele. The values of the Pearson correlation coefficient (ρ) for each case (B)–(E) are shown.

With allelic regulation and stochastic partitioning during mitosis, our simulations clearly showed that unlike the NANOG profile the GFP distribution features two distinct modes even in the absence of transcriptional noise (Figure 4A). More importantly, the heterogeneity associated with GFP is more pronounced (coefficient of variation or CV = 1.0) than that of the actual NANOG distribution (CV = 0.74). Thus, caution should be exercised when examining data from experiments with stem cell lines featuring knocked-in reporter genes.

We further tested if the read-out of this reporter system was reliably reflecting the dynamic expression of the native gene under different assumptions. As expected, when the same t_1/2_ was assumed for both NANOG and the reporter protein (Figure 4B), their levels were perfectly correlated (Pearson product-moment correlation coefficient ρ = 1.00) if the reporter gene was inserted in both alleles. In the case of a single-allele knock-in (Figure 4C) however, a subpopulation of the stem cells (NANOG^high^/GFP^low^) could not be correctly reported due to the effect of allelic regulation (ρ = 0.66).

The situation of NANOG and a reporter protein having the same t_1/2_ is unlikely when GFP (or several of its variants) is considered. This results in non-matching profiles for the two proteins (ρ = 0.89) even when both alleles carry the fluorescent marker gene (Figure 4D). Reporter systems used in practice carry the reporter gene in one of the two targeted alleles with different half-lives for the native and reporter gene products. Not surprisingly, cells with insertion of the reporter gene in one allele showed the lowest correlation (ρ = 0.60) between the expression of reporter and NANOG with different t_1/2_ (Figure 4E). Specifically, GFP^high^ mESCs were also NANOG^high^ but a portion of NANOG^high^ mESCs fell within the GFP^low^ region. These cells may be misconstrued as autofluorescent or similar to isotype controls. Additionally, mESCs exhibit heterogeneity in reporter and NANOG levels but the reporter read-out does not vary linearly with the NANOG expression level as in Figure 4B.

**Figure 5:**
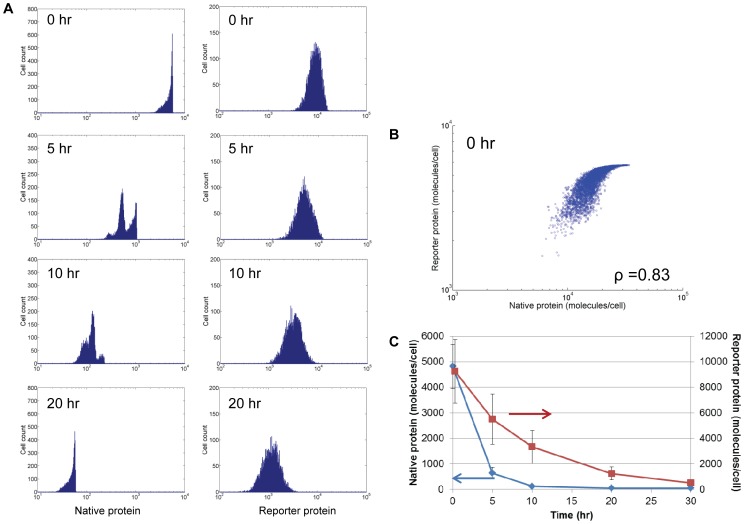
Correlation between endogenous protein and reporter levels in a single-allele reporter system in the absence of allelic control. (A) At t = 0, the expression of native and reporter genes from both alleles is shut down for cells at equilibrium state. Distributions at subsequent times are shown for the endogenous and reporter proteins. (B) Endogenous protein-reporter protein scatter plot corresponding to t = 0 of (A). The Pearson correlation coefficient is also shown. (C) Average protein levels are plotted over time. Values are shown as mean±st.dev.

Since allelic regulation is not universally applicable to stem cell genes (e.g. *pou5f1* (Oct4)), we also analyzed a more general case for an endogenous gene X not subjected to this mode of expression control. When this gene is expressed at steady state, its level correlates qualitatively with the level of the corresponding reporter in a straightfoward manner. We therefore considered transient expression of gene X as in the case of pluripotency markers at the onset of differentiation or upon treatment with transcriptional inhibitors [35]. For this purpose, transcription of the native and reporter genes was turned off in the PBE model (see **Materials and Methods**) and the temporal evolution of the respective protein distributions was tracked (Figure 5A). Without allelic regulation (Figure 5B), the single-allele reporter system displayed a tighter correlation between the expression of the reporter and endogenous proteins (ρ = 0.83 vs. ρ = 0.60 in Figure 4E) and was maintained over 20 hours (ρ∼0.82; time equal to t_1/2_ of reporter). Hence, the reporter signal qualitatively still reflected the endogenous protein level. However, the relative decrease in reporter protein over time lagged the native protein reduction significantly (Figure 5C) although both eventually converged to distributions with lower mean values (Figure 5A).

Taken together, our data demonstrate that in stem cell lines expressing reporter genes from the Nanog gene locus, the reporter protein level is not reflective of the endogenous NANOG protein. Actually, stem cells carrying the reporter gene in one target allele are commonly utilized in research today. These cells exhibited the highest divergence in the profiles of the native protein and its surrogate reporter. Therefore, the effects of allelic regulation should be accounted for when interpreting relevant data. Lastly, differences in the reporter and target protein half-lives contribute to disparate profiles of transiently expressed genes regardless of whether the reporter gene is knocked in one or both alleles even when there is no allelic regulation.

### Stem cells with single Nanog allele deletion maintain a pluripotent subpopulation

We showed that normal cells having inactive both Nanog alleles (e.g. type ‘4’ cells in Figure 1C) eventually reconstitute a heterogeneous population featuring cells with high Nanog preventing commitment. Thus, we asked the question: How does the deletion of one copy of *nanog* affect the capacity of mESCs to maintain a pluripotent state given the allelic regulation of the gene? This segment of our work was motivated by conflicting findings in experiments utilizing Nanog mutant cells. Hatano et al. [5] observed that *Nanog* ^+/−^ mESCs readily differentiate in spite of being cultured with LIF. Others also reported that suppression of Nanog leads to reduced expression of other pluripotency markers [7] and induces differentiation [6] in hESCs. Still, Chambers et al. [16] in an elegant study reported that *Nanog* ^+/−^ and *Nanog* ^−/−^ mESCs continue to self-renew in the absence of differentiation stimuli and form colonies with similar morphology as pluripotent mESCs concluding that Nanog acts to safeguard pluripotency but is not an indispensable factor.

**Figure 6:**
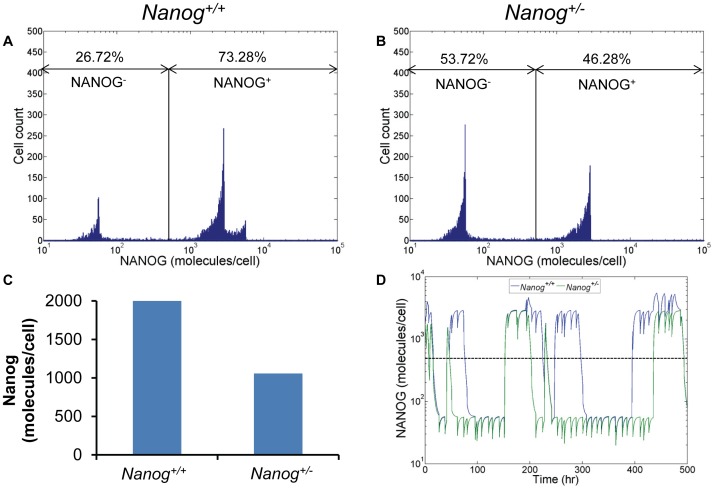
Prediction of the effect of single allele deletion on Nanog expression. Nanog expression distribution in (A) *Nanog* ^+/+^ and (B) *Nanog* ^+/−^ mESCs. The fractions of NANOG-positive and -negative cells are also shown. (C) Average NANOG expression level of *Nanog* ^+/+^ and *Nanog* ^+/−^ cell populations calculated from the distributions in (A) and (B). (D) Comparison of Nanog fluctuations in single *Nanog* ^+/+^ (blue) and *Nanog* ^+/−^ (green) mESCs. Dashed line indicates the threshold between NANOG^+^ and NANOG^−^ cells.

To that end, the PBE model was modified by turning off the expression of Nanog from one allele to account for the *Nanog* ^+/−^ genotype (see **Materials and Methods**). When comparing the distribution of NANOG in wild-type and mutant mESC populations, the latter cells still exhibited NANOG^+^ mESCs. However the fraction of NANOG^+^ mESCs dropped from approximately 73% for normal mESCs to almost 46% for *Nanog* ^+/−^ cells (Figures 6A–B). This was concomitant with an increase in the heterogeneity of the population (CV = 0.74 and 1.08 for Figures 6A and 6B, respectively). It should be noted that in flow cytometry assays the line separating the NANOG^−^ and NANOG^+^ cells (500 molecules/cell here) between the first and second/third peaks is determined based on appropriate isotype controls. Shifting the line within this region did not alter the fractions of cells significantly. The average NANOG amount per cell was almost half in the *Nanog* ^+/−^ mESC population than in normal mESCs (Figure 6C) in line with western blot results by Hatano et al. [5]. Our findings show that deletion of one Nanog allele does not simply reduce NANOG uniformly for all mESCs but modulates NANOG heterogeneity directly.

Examination of the NANOG fluctuations in single cells further illustrated this effect (Figure 6D). Compared to wild-type mESCs, *Nanog* ^+/−^ cells had a lower chance of switching back to a NANOG^+^ state due to allele deletion. In fact, almost 60% of wild-type mESCs with both alleles in the ‘off’ state switched on at least one allele within five cell cycles and the steady-state mESC population was reconstituted within 100 hours (see Figure 1C). In contrast, the corresponding fraction of *Nanog* ^+/−^ mESCs was only 43%. Nonetheless, the higher fraction of NANOG^−^ cells indicates that loss of one Nanog allele results in a commitment-permissive state. Thus, *Nanog* ^+/−^ cells remain pluripotent in the absence of differentiation signals but over half of the population will promptly differentiate upon induction with appropriate factors.

## Discussion

Nanog is a core pluripotency transcription factor influencing the decision of stem cells to self-renew or differentiate. The recent demonstration that Nanog is allelically regulated in mESCs calls for reexamination of findings about the role of Nanog on the maintenance of the pluripotent state and the propensity of stem cells for commitment to particular lineages. It also provides a new vista for the interpretation of data from engineered stem cell lines with reporter genes knocked in the Nanog gene locus. Allelic regulation of Nanog expression has not been demonstrated experimentally in human stem cells but we surmise that work in this direction is in progress, especially given that this mechanism is plausible when analyzing pertinent hESC data. With these considerations in mind, we developed a PBE model taking into account the allelic regulation of Nanog in conjunction with the asynchronous cell proliferation and gene expression dynamics. Besides recapitulating the experimental findings of Miyanari et al. [20], our results clearly demonstrate that any of the four mESC types under routine maintenance conditions (LIF and serum) gives rise to mESC populations with the same heterogeneity with respect to Nanog expression. This is particularly significant because Nanog coordinates multiple genetic programs during development and reprogramming and potentially regulates heterogeneity [15], which translates to variable proclivity for self-renewal or commitment among cells of the same population. Indeed, a subpopulation of self-renewing cells residing at a state with lower Nanog content is primed for specification upon induction with suitable factors. In its current form, the framework does not consider differentiation but work in this direction is underway [36].

The Nanog distribution in mESC populations at equilibrium features three peaks corresponding to types ‘1’ (biallelic), ‘2’+‘3’ (monoallelic) and ‘4’ (both alleles being inactive) seemingly contrasting previous reports of a bimodal NANOG (GFP) distribution in mESC and hESC lines with the *gfp* expressed from the Nanog locus [17], [18]. One may argue however that in a flow cytometry assay the lowest Nanog content (type ‘4’) peak ‘L’ would overlap most likely with the isotype (or autofluorescence) control and therefore the cells would be considered as NANOG^−^ akin to the LN mESCs [18] and to hESCs [23]. Additionally, sorted LN cells reconstitute the original bimodally distributed population of LN/HN cells upon subculturing similar to our results with a starting population of type ‘4’ mESCs. We also showed that gene expression noise causes the peaks ‘M’ and ‘H’ (NANOG^+^ cells) to merge yielding a bimodal profile. The existence of three states based on Nanog expression for mESC populations has been recognized in a recent study with the introduction of a middle Nanog (MN) state [22]. Thus, discrepancies between the present and other studies regarding the NANOG profile of self-renewing stem cell populations appear to be largely reconciled.

Nevertheless, the underlying determinants of the NANOG distribution are significantly different. The existence of the LN population was explained earlier through the concept of excitability in a GRN of Nanog with Oct4 and Sox2 [18]. A transient low expression of Nanog (LN) ensues when the GRN featuring a negative feedback loop is perturbed by transcriptional noise. Others have also employed the same three-transcription factor GRN with modifications to study NANOG variability [19] concluding that oscillations or noise in Nanog expression leads to a similar two-peak profile. Gene expression noise is a major determinant of the distribution of NANOG in stem cells [23]. Our model provides alternative mechanisms driving the emergence of the NANOG heterogeneity observed in mESC cultures. The bimodal distribution of Nanog emerged in our analysis by considering allelic regulation, asynchronous cell proliferation, and stochastic partitioning of NANOG with or without transcriptional noise in a single-gene model. Allelic control of Nanog expression has been elegantly demonstrated in mouse embryos and mESCs [20] supporting our findings. Yet, practical methods for controlling noise in cellular processes are still lacking. This leaves open the question of whether (and if so how) allelic modulation of gene expression acts in concert with one or more excitable GRNs under transcriptional noise to promote diversity in isogenic stem cell populations.

Our framework also provides a rationale regarding the stability of the LN state. Sorted HN mESCs (GFP^+^) give rise to a population with a lower fraction of LN mESCs (GFP^−^; 7%) than the HN group (38%) of sorted LN mESCs cultured for the same period (48 hours) [18]. Supported by an excitable GRN model, this observation led to the conclusion that the LN state is unstable with frequent transitions to the HN, whereas the latter state is stable and conversions to the LN state are rare. Potential discrepancies between actual Nanog expression and GFP signal aside, we also observed that a number of type ‘4’ cells are classified as NANOG^high^ cells especially if they have just exited the state in which both *nanog* alleles were active. Cells with both alleles recently inactivated, require longer time to deplete their NANOG reserve whereas those exiting this state build their protein content faster. Therefore, the experimentally observed dynamics of the HN and LN states are supported by our model mainly as a result of allelic regulation of Nanog.

Unlike other reports employing GRNs, the Nanog expression dynamics in this study were described by a single-gene model with “on” and “off” states. This approach was advantageous in two ways: First, adoption of a GRN model necessitates assumptions about the structure of the network. Structures of GRNs involved in stem cell fate decisions are not well-established. For example, Navarro et al. [37] recently reported that Nanog activity is autorepressive and independent of Oct4/Sox2 unlike GRNs utilized in previous studies. Second, GRN models typically involve several parameters which are currently impossible to determine through experiments. Although we utilized a single-gene expression model, the PBE framework is amenable to the incorporation of GRNs, especially as more information comes to light from research on the interactions of Nanog with other partners.

It should be noted that culture conditions affect the relative portions of stem cells in different Nanog states. Mouse ESCs maintained in medium containing serum and LIF achieve equilibrium with fractions reflected by 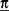 [20]. We considered this as our model system since mESCs are commonly cultivated with LIF and serum. However, the same analysis can be carried out for other conditions. For example, growing mESCs in 2i leads to a significant enhancement in biallelic *nanog* expression thereby changing the relative portions of different subpopulations at equilibrium (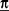) [20]. An analysis of the Nanog distribution in mESCs under different culture conditions has been reported [22] without considering explicitly allelic gene regulation. The corresponding model is based on the calculation of a one-dimensional ‘potential energy’ function representing the ‘barrier’ for cells moving between intermediate states. Others [38] have also modeled the transition of stem cells between attractor states through a quasi-potential energy function in an epigenetic landscape introduced by Waddington [39].

The allelic control of Nanog expression calls for closer scrutiny of stem cell lines carrying reporter genes such as GFP and its variants. Use of such lines is warranted on the premise that the reporter signal can serve as a surrogate closely matching the expression of a protein from the same genetic locus. Our simulation results illustrate that the reporter signature varies drastically depending on whether its gene is inserted in one or both target alleles, even under the assumption of equal t_1/2_ for the reporter and native gene products. Thus, stem cell lines intended for monitoring genes subjected to allelic regulation should have the reporter gene inserted in both alleles. Obviously, this entails practical considerations as such construction is significantly more cumbersome than that of single-allele knock-ins. Reporter genes are inserted in the targeted locus usually by homologous recombination which is a notoriously inefficient process although certain modifications may enhance its efficiency [40]–[42]. Because allelic control of expression is not universal, single-allele residing reporter gene cell lines may be sufficient for monitoring genes not subjected to this mechanism.

Still, an important factor in monitoring gene expression via a reporter surrogate is the difference in the kinetics (typically exemplified by the t_1/2_) for net production of the native and reporter proteins translating to non-matching profiles. This disparity may be partially alleviated with the use of proper fast-degrading (destabilized) reporter variants [43], [44] but should not be overlooked as it is fundamental for proper interpretation of pertinent data. In fact, the PBE model described here can be used to back-calculate the actual expression profile of the protein of interest from reporter distributions. The process entails the estimation of parameter values for reporter production and switch on/off rates. The same values will apply to the native protein distribution due to the matching regulation by virtue of sharing the same chromatin site. If the gene is allelically regulated, transition rate parameters can be obtained, for example, from immunocytochemistry/RNA FISH or single-cell allele-specific RT-PCR data. Other PBE parameters can be determined as we described previously [23]. The cell doubling time (T_d_) can be measured in cell culture experiments and the t_1/2_ values of the reporter and the native protein also can be obtained through well-established methods [45]. With this information available, the PBE model can be run to generate the actual profile of the target protein. This approach is straightforward when the distributions of the reporter/protein are time-invariant. The same methodology can be applied to temporally fluctuating distributions but requires detailed knowledge of the mechanism(s) governing the evolution of reporter and protein production. Additional information may also be necessary, for example, in differentiation experiments where the expression of pluripotency and lineage-specific markers changes with time. A major challenge in these experiments is the identification of appropriate single-cell functions describing the dynamics of stem cell commitment. The timing of the measurements also becomes relevant since our results show that the decay in reporter protein with a longer t_1/2_ lags that of the target protein when both genes are not actively transcribed (Figure 6C). Analogous results can be obtained for a reporter and its target protein when the transcription of both is turned on under proper conditions.

The model also shines light on whether Nanog^−^ stem cells in a self-renewing population may regain or lose irreversibly their pluripotent status. We demonstrated that starting with a group of wild-type mESCs having both *nanog* alleles ‘off’, over 60% of them switch on at least one within five cell cycles in non-differentiating conditions. In the same interval, *Nanog* ^+/−^ mESCs with their *nanog* allele initially inactive transition to a population where 43% of the cells are NANOG^+^. Thus, even NANOG^−^ mESCs can self-renew and reestablish a NANOG^+^ population in agreement with previous studies [2], [5], [16], [18]. The framework in its present form helps to predict if a cell within an ensemble will continue to self-renew or commit to a particular fate if exposed to differentiation stimuli. Such prediction entails the knowledge of a Nanog content threshold for differentiation-preventive vs. -permissive stem cell self-renewal.

Nonetheless, further research is needed to address a distinct question, i.e. to which lineage a differentiation-primed NANOG^−^ stem cell will convert. The lineage propensity of cells with low or no Nanog expression is debatable. According to Mitsui et al. [2], *Nanog* ^−/−^ mESCs primarily express markers of parietal and visceral endoderm, whereas others [5] showed that *Nanog* ^+/−^ cells express genes of the three embryonic germ layers. Their results suggest a Nanog content-dependent differentiation with extraembryonic endodermal fates favored in the absence of Nanog and mesodermal, endodermal and ectodermal progeny being generated from cells with Nanog content gradually decreasing by 0–50% compared to pluripotent state ESCs. Mouse ESCs at the LN state cultured in neuronal differentiation medium may still revert to the HN state albeit at a low fraction (16%) [18]. Since Nanog interacts with multiple partners in pluripotency and differentiation programs [4], long-term residence of stem cells in the NANOG^−^ state may eventually lead to differentiation, even with small perturbations in their microenvironment. For instance, no changes are evident in transcriptional regulatory network partners of Nanog until at least three days after its depletion [15]. Longer-term expansion of *Nanog* ^−/−^ mESCs without loss of their pluripotency has also been reported with variable degrees of success [5], [16]. Thus, the kinetics of NANOG^−^ stem cells undergoing differentiation vs. self-renewal and the balance with the NANOG^+^ cells remain to be elucidated.

The time span between the complete decline in Nanog content and loss of pluripotency is also an illustration of the multiscale nature of stem cell fate specification [15], [16]. We view that models for stem cell populations should consider together subcellular (e.g. regulation of pluripotent/differentiation marker expression, signal transduction), intercellular (e.g. paracrine signaling) and population-wide processes (e.g. cross-talk among subpopulations with distinct phenotypes). These phenomena are not only innate to the stem cell niche and major determinants of fate decisions but also transpire over markedly different time scales. Multiscale PBE approaches afford coping with the multiple temporal/spatial scales of stem cell processes. In the present study, rapidly fluctuating gene expression dynamics were combined with significantly slower events such as cell proliferation and allelic regulation. At the same time, there is flexibility in the implementation of models for deterministic or stochastic phenomena such as the transcription and allelic switching of *nanog*.

In conclusion, the stochastic PBE model developed in this study is aligned with the experimental findings on the allelic switching of Nanog expression and the heterogeneity of cells with single *nanog* allele deletion. Our results illustrate that allelic regulation is pivotal for the observed heterogeneity of ESCs with respect to Nanog content. The same mechanism may very likely influence the diverse presentation in stem cell populations of other markers (e.g. Oct4, Stella, Sox2, Rex1), which are intricately connected to the expression of Nanog. Lastly, the significant problems linked to the use of reporter cell lines for monitoring Nanog (or other genes) are portrayed. The PBE framework provides a platform for addressing these issues in practice and may serve as a tool complementing experiments to gain a deeper understanding of stem cell population heterogeneity in connection with fate specification. These outcomes will accelerate the development of efficient differentiation and reprogramming methods for the generation of therapeutically useful progeny.

## Materials and Methods

### Calculation of transition probabilities and rates

The transition probabilities 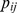 for a cell switching from state i to state j can be calculated considering (a) the limiting distribution 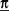 and (b) information regarding the numbers of cells shuttling between these states. Such information is available per cell cycle (unit of time) in the report by Miyanari et al. [20] and as indicated in Figure 1. For instance, 12% of the total mESC population shuttles between states 1 and 2. The percentages of cells switching from i to j and from j to i states are assumed to be equal. Then, detailed balances can be written, i.e.
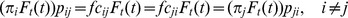
(A1)where 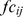 represents the fraction of the cell population transitioning from state i to j. For instance, 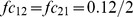 yielding
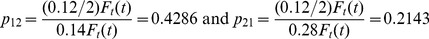
given that 14% and 28% (elements 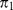 and 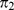) of the total population 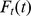 are in states 1 and 2, respectively. The other transition probabilities are calculated in the same fashion noting that 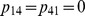 since states 1 and 4 are not linked directly. Moreover, 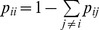.

The transition rates 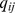 for cells switching from state i to j are defined as 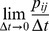 [46]. Here, the data for calculation of the transition probabilities refer to a single cell cycle time T_d_ (unit time of observation) and thus, the transition rates are approximated as 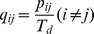. Moreover, 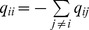 holds true based on transition matrix properties.

The system of differential equations (Equation 4) describing the temporal evolution of the subgroups of mESCs exhibiting distinct allelic expression of Nanog in terms of cell numbers (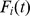) can be re-written based on the corresponding percentages 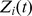:
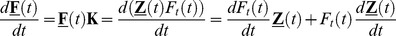
(A2)


This results in the following expression:
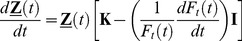
(A3)where 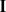 is the (4x4) identity matrix and because
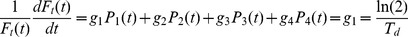
(A4)then,
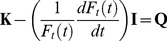
(A5)


This yields Equation 6 in the main text. This can also be written as:
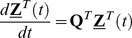
(A6)At steady state there is a non-trivial solution since the rank of 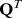 is 3 and the corresponding vector of the null space is: 
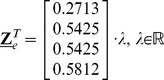
. The non-trivial solution is subjected to the constraint: 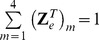. The results are consistent with the findings from mESC experiments yielding 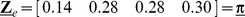.

### PBE model functions

The master PBEs (Equation 12) contain the following functions:

- Growth rate function 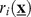: The dynamics for r_i_(N_1_) and r_i_(N_2_) have been described in the main text (Equations 7–10). The growth rate for cell size was taken as: 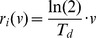 where T_d_ is the mESC doubling time. The same growth expression was considered for all four types of mESCs.
  - Dividing rate function 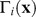: Its derivation is based on the assumption that the size of dividing cells follows a Gaussian distribution as shown by Tzur et al. [29] and this distribution was assumed for all types of mESCs.
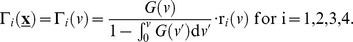


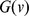
 is a Gaussian distribution with mean μ and standard deviation σ. The values of the parameters were calculated previously [23] and are listed in Table 2.
  - Partitioning function 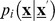: The partitioning function is a β distribution [29] and elements 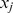 (i.e. 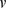, 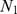 or 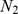) of the state vector 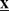 are partitioned independently of each other. All types of mESCs have the same partitioning function:
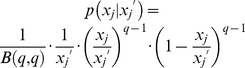
 with 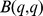 being a symmetric beta distribution. Obviously, 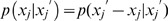 and the total 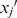 is conserved during division. The value of parameter q is also listed in Table 2.

**Table 2:** PBE model parameters [23].

Parameter	Mean ± St. dev.
μ	0.465±0.043
σ	0.104±0.022
q	39±2.291

### Description of PBE solving algorithm

A schematic of the Monte Carlo (MC) algorithm for obtaining numerical solutions of the PBE model has been described previously [23] and is shown in Figure S2. In addition, we detail below the selection of a specific event interrupting quiescence (i.e. cell division or switching between allelic Nanog expression patterns).

For this purpose, matrix 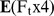 was set up with F_t_ rows (i.e. equal to the total number of cells) and 4 columns for the four mESC states. The n^th^ row corresponds to a cell from the i^th^ subgroup and contains the pertinent transition rates 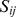
(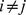) and proliferation rate 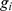
(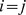). Given a random number *ran2* from a uniform distribution, we identify: (a) The k^th^ cell which will disrupt quiescence, and (b) whether this cell will divide or switch to a different state:
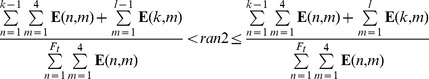
or (if 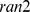 falls between two successive cells in the matrix **E**)
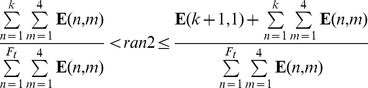



The above inequalities allow for the identification of the k^th^ mESC, which is at the i^th^ state. If 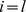 then this cell divides, otherwise it switches from i^th^ to the l^th^ state. Initially the algorithm is a constant volume MC as the cell population size increases up to a limit (typically 10,000 cells). Subsequently, the algorithm becomes a constant number MC [23] with the daughter cells replacing the mother cell and another randomly selected cell (see Figure S3). Simulation programs were written in FORTRAN. MATLAB (Mathworks, Natick, MA) was utilized mainly for post-processing of results.

### PBE model without allelic regulation (endogenous gene X and reporter gene)

The PBE model was modified to simulate the temporal evolution of an endogenous gene X and a reporter gene when both are not subjected to allelic regulation. The off-diagonal elements of matrix 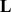 were set to zero because there is no allelic switch and all cells in the population belong to (sub)group ‘1’. Initially, all cells in the ensemble express the reporter and X genes. Subsequently, expression of X and the reporter was turned off by using the “off” state values for the parameters in the single-gene model.

### PBE model modification for deletion of single Nanog allele

We assumed without loss of generality that Nanog allele 1 was deleted in *Nanog* ^+/−^ mESCs by setting and maintaining the gene expression from allele 1 in the “off” state for the duration of the simulation as shown in Table S1. The expression dynamics and pertinent parameters for the functional allele (allele 2) remained the same as described in the model development paragraph.

## Supporting Information

Figure S1Effect of the production rate parameter value 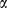 on the expression profile of NANOG. The distribution of NANOG is shown for the parameter set at (A) 1000 molecules/hr or 20% below (B) or above (C) this value. The corresponding single-cell signatures of expressed NANOG are shown in (D)–(F). The degradation rate 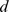 was kept constant.(TIF)

Figure S2 Schematic of the Monte Carlo algorithm implemented for obtaining numerical solutions of the PBE model.
(TIF)

Figure S3Illustration of event selection based on event rate matrix 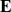. Here **E(10,4)** contains 10 cells (rows) and each cell is associated with probabilities for proliferating or transitioning to other patterns of allelic regulation of Nanog. The difference in the color between neighboring elements is the event rate normalized to the total rate of all the events (color bar). A random number from a uniform distribution (e.g., *ran2* = 0.633) is used to determine which cell and which type of event (division or allelic switching) will occur at the end of the current interval of quiescence.(TIF)

Table S1 Summary of Nanog allele state for each subtype of *Nanog* ^+/−^ mESCs (allele 1 deletion). The mESC types ‘1’–‘4’ are as shown in Figure 1.
(DOCX)
