# Supplementary figures and images for: Distinct Allelic Patterns of Nanog Expression Impart Embryonic Stem Cell Population Heterogeneity
Source: PLoS Comput Biol. 2013 Jul 11;9(7):e1003140. doi: 10.1371/journal.pcbi.1003140 (PMC3708867; doi:10.1371/journal.pcbi.1003140)

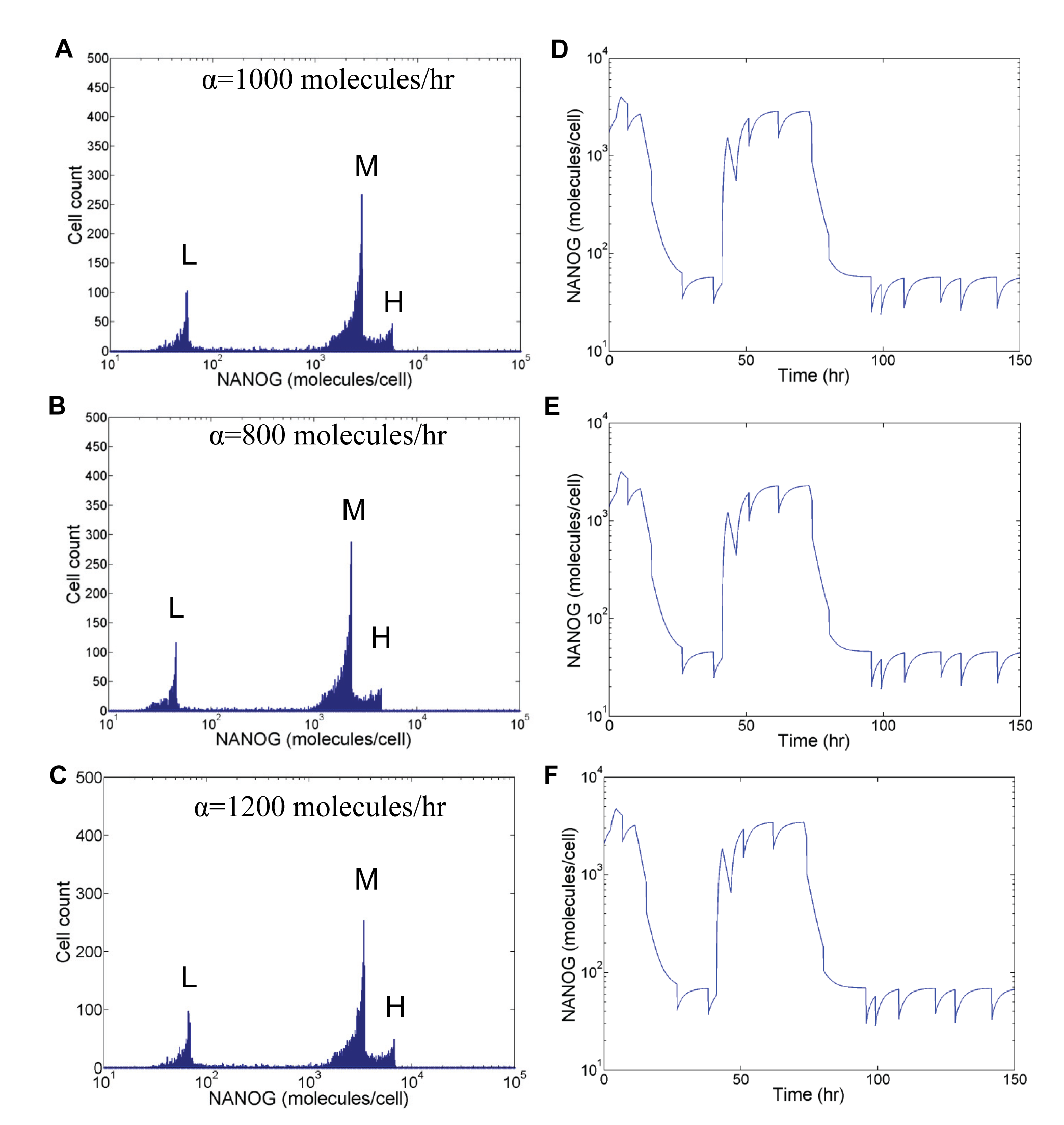

Supplement: Figure S1 — Effect of the production rate parameter value on the expression profile of NANOG. The distribution of NANOG is shown for the parameter set at (A) 1000 molecules/hr or 20% below (B) or above (C) this value. The corresponding single-cell signatures of expressed NANOG are shown in (D)–(F). The degradation rate was kept constant. (TIF) [file pcbi.1003140.s001.tif]

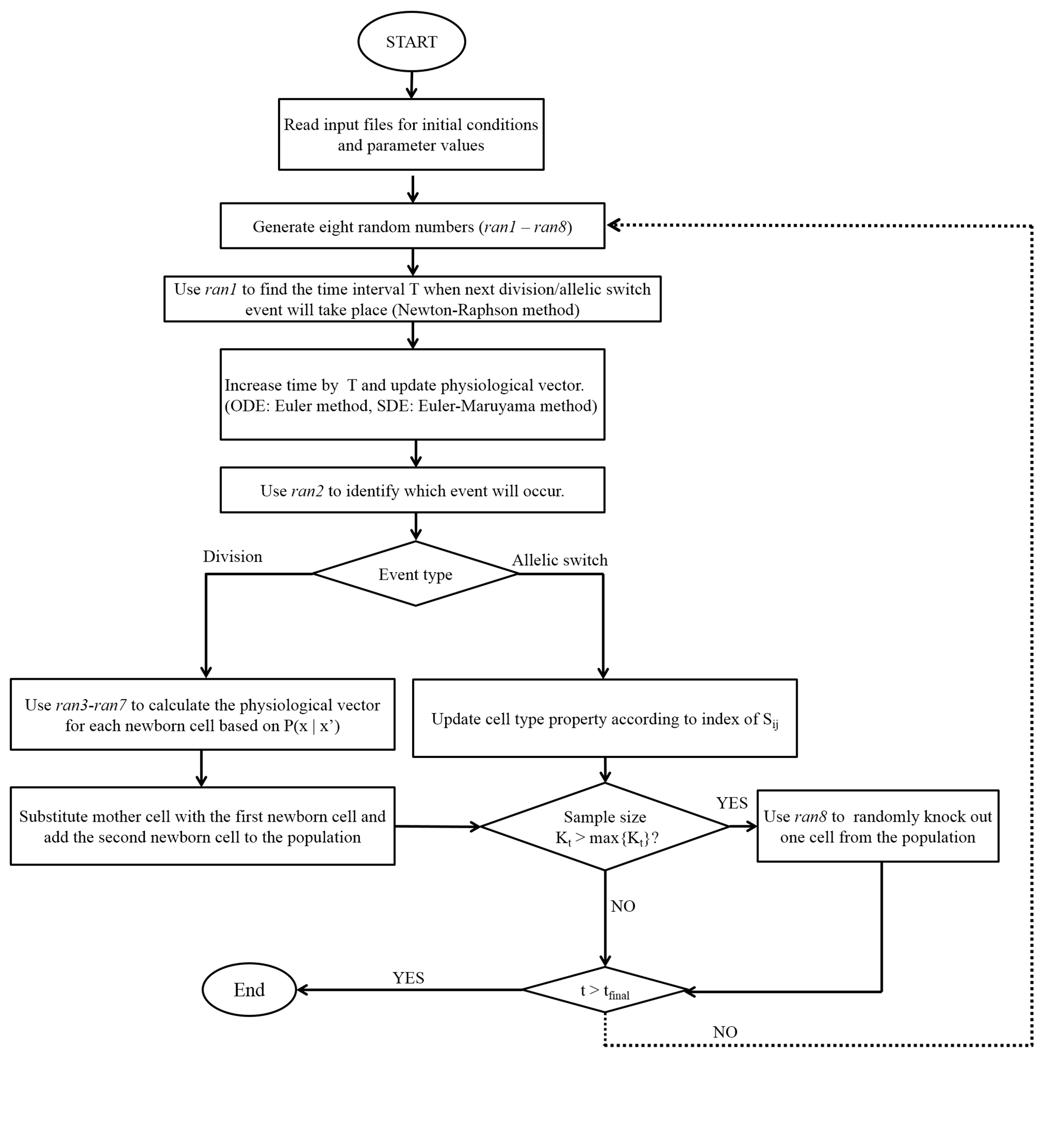

Supplement: Figure S2 — Schematic of the Monte Carlo algorithm implemented for obtaining numerical solutions of the PBE model. (TIF) [file pcbi.1003140.s002.tif]

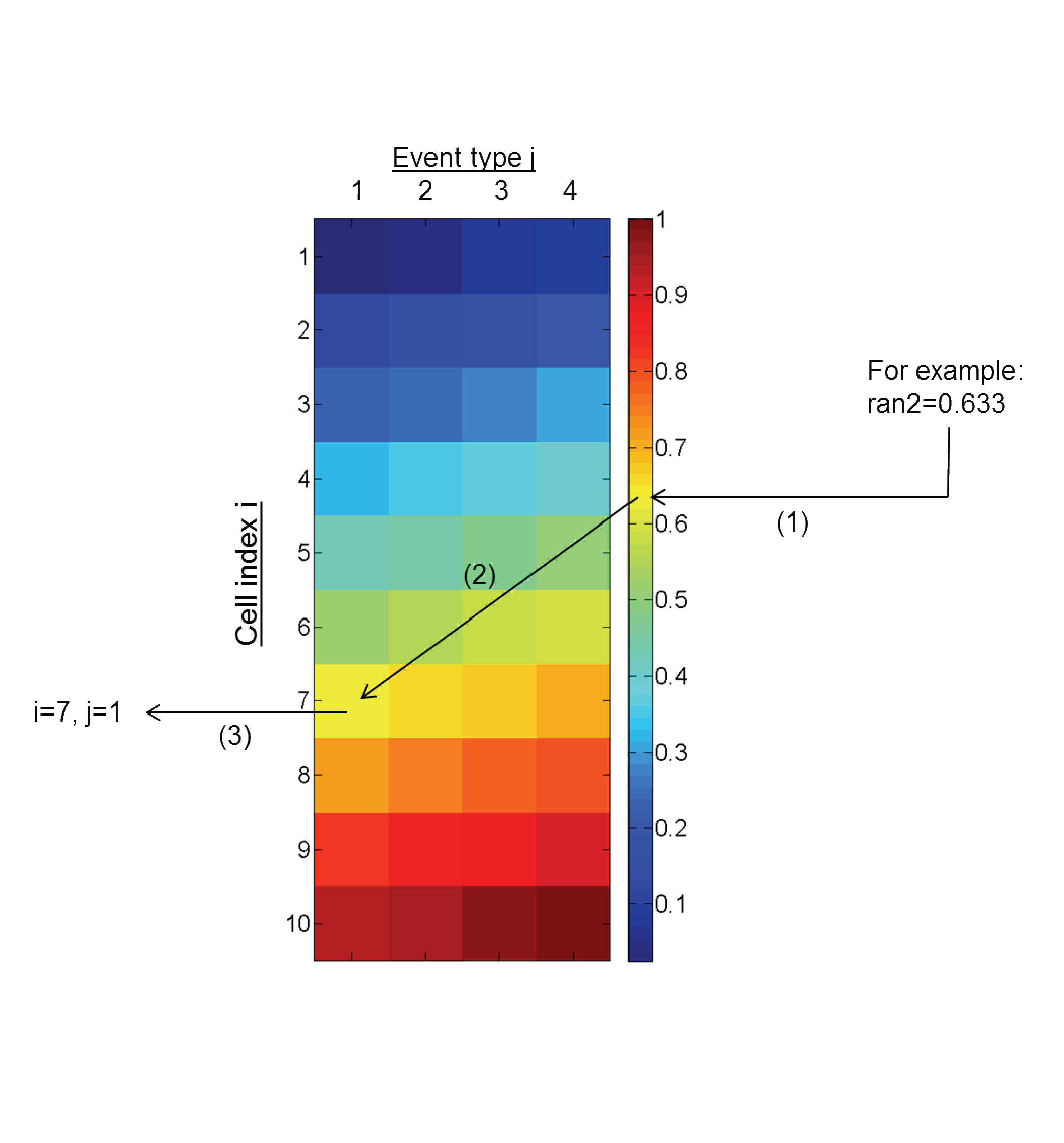

Supplement: Figure S3 — Illustration of event selection based on event rate matrix . Here E(10,4) contains 10 cells (rows) and each cell is associated with probabilities for proliferating or transitioning to other patterns of allelic regulation of Nanog. The difference in the color between neighboring elements is the event rate normalized to the total rate of all the events (color bar). A random number from a uniform distribution (e.g., ran2 = 0.633) is used to determine which cell and which type of event (division or allelic switching) will occur at the end of the current interval of quiescence. (TIF) [file pcbi.1003140.s003.tif]
