# Supplementary material for: Distinct Allelic Patterns of Nanog Expression Impart Embryonic Stem Cell Population Heterogeneity
Source: PLoS Comput Biol. 2013 Jul 11;9(7):e1003140. doi: 10.1371/journal.pcbi.1003140 (PMC3708867; doi:10.1371/journal.pcbi.1003140)
Supplement: Table S1 — Summary of Nanog allele state for each subtype of Nanog +/− mESCs (allele 1 deletion). The mESC types ‘1’–‘4’ are as shown in Figure 1. (DOCX) [file pcbi.1003140.s004.docx]

| Cell type | NANOG allele 1 | NANOG allele 2 |
| --- | --- | --- |
| 1 | Off | On |
| 2 | Off | Off |
| 3 | Off | On |
| 4 | Off | Off |
